# Atypical onset of diabetes in a teenage girl: a case report

**DOI:** 10.1186/1757-1626-1-425

**Published:** 2008-12-30

**Authors:** Cristina Maria Mihai, Doina Catrinoiu, Ramona Mihaela Stoicescu

**Affiliations:** 1 Pediatric Department for Diabetes, Nutrition, and Metabolic disorders in children, "Ovidius" University Constanta, Faculty of Medicine, Constanta County Emergency Hospital, 145 Tomis Blvd, 900591, Constanta, Romania; 2 Adult Department for Diabetes, Nutrition, and Metabolic disorders in adults, "Ovidius" University Constanta, Faculty of Medicine, Constanta County Emergency Hospital, 145 Tomis Blvd, 900591, Constanta, Romania; 3 Laboratory Department, "Ovidius" University Constanta, Faculty of Medicine, Constanta County Emergency Hospital, 145 Tomis Blvd, 900591, Constanta, Romania

## Abstract

**Background:**

Chorea, hemichorea-hemiballismus and severe partial seizures may be the presenting feature of nonketotic hyperglycemia in older adults with type 2 diabetes, but cases in children with type 1 diabetes are rare, since the most easily recognized symptoms of type 1 diabetes in children are secondary to hyperglycemia, glycosuria, and ketoacidosis.

**Case presentation:**

A previously healthy 15-year-old girl presents with sudden onset of right-sided chorea. Brain CT did not detect any abnormal density areas. A T1-weighted image of brain MRI was normal. Investigations revealed hyperglycemia with absent ketones and normal serum osmolality. Achievement of normoglycemia with insulin therapy determined the involuntary movements to regress completely within a day. The direct effect of hyperglycemia could be the pathogenesis of the chorea in our patient. Severe hyperglycemia without ketosis at the clinical onset of insulin-dependent diabetes mellitus (type 1) has been reported in children and adolescents, but nonketotic hyperglycemia is an unusual cause of chorea-ballismus in children, and chorea-ballismus is also a rare manifestation of primary diabetes mellitus.

**Conclusion:**

The importance of clinical evaluation, laboratory testing and neuroimaging for the differential diagnostics of chorea is emphasized.

## Background

Chorea is a clinical symptom characterized by: spontaneous involuntary movements, muscular weakness and incoordination of voluntary movements and can be classified as idiopathic or hereditary or symptomatic/secondary. Chorea or ballismus can be caused by a wide variety of degenerative, metabolic or vascular disorders affecting the basal ganglia: metabolic diseases, hypoxic-ischemic events, vascular disorders, structural abnormalities, trauma, drugs and toxins, infections and inflammatory immunological diseases (rheumatic fever – Sydenham's chorea, systemic lupus erythematosus). Chorea has been frequently associated with lesions in the basal ganglia, and in the subthalamic nucleus [1,2].

Neuroimaging studies can demonstrate a lesion of the subthalamic nucleus or in other different subcortical structures.

Hyperglycaemia-induced hemiballismus-hemichorea is an uncommon movement disorder probably related to vascular insult of the basal ganglia in patients with poorly controlled diabetes. When these movements are confined to one side of the body, i.e. hemichorea-hemiballismus (HC-HB), lesions in the contralateral subthalamic nucleus and pallidosubthalamic pathways are usually present. Recognition of the association of these neurological abnormalities and non-ketotic hyperglycemia is important because the correction of the underlying hyperglycemia will lead to rapid improvement.

## Case presentation

A 15 year old girl was brought to the emergency department with a history of sudden onset of paresthesia, involving initially the right leg, then, the right arm, the face and, subsequently the entire right side of the body, increasing during activity (walking) and ceasing during sleep, of two days duration. The patient described this as „the feeling of pins and needles“. There was no previous history of fever, headache, and neurological illnesses. She did not receive any medication. She was non-smoker. Clinical examination revealed a normal developed young girl, with normal memory, speech and orientation, with normal physical exam.

The preliminary impression of the emergency staff was of a functional disorder, and the patient was discharged home, with the recommendation to take calcium and magnesium supplements, being well known that the paresthesia of the mouth, hands, and feet is a common, transient symptom of the related conditions of hyperventilation syndrome and panic attacks. The patient was called back for a formal neurologic assessment scheduled for the next day. On the same day, the patient came back, with repeated involuntary movements involving her right side (foot, arm and face). She was admitted in the Intensive Care Unit and a diagnosis of Hemichorea-Hemiballismus (HC-HB) was made.

Hypotonia of muscles with normal power and normal deep tendon reflexes were present symmetrically. Her blood pressure was 110/55 mm Hg. Keyser Fleischer ring was absent at slit lamp examination.

This clinical symptom of spontaneous hemichorea was considered secondary to a poststreptococcal neurological disease, Sydenham's chorea, but was not associated with other clinical features of rheumatic fever (carditis, arthritis, erythema, rheumatic nodules) and neuropsychological features (dysarthria and emotional disorders). Also, the choreiform movements are rather more continuous in chorea Sydenham than paroxysmal as observed in the described case and the serum anti-streptolysin-O titre was < 200 IU.

Also, the possibility of continuous focal seizures (epilepsia partialis continua [EPC]) causing unilateral movements and CNS involvement (the contralateral basal ganglion, the thalamus and the subcortical areas) was considered. As a result, electroencephalography and brain CT and MRI scanning were scheduled.

**Figure 1:**
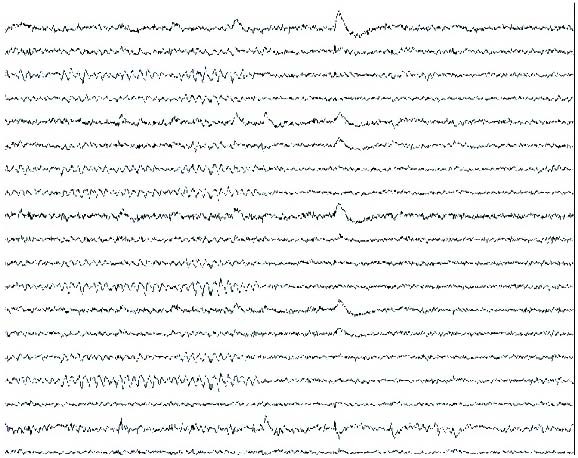
**Awake EEG, no chorea-ballismus**.

**Figure 2:**
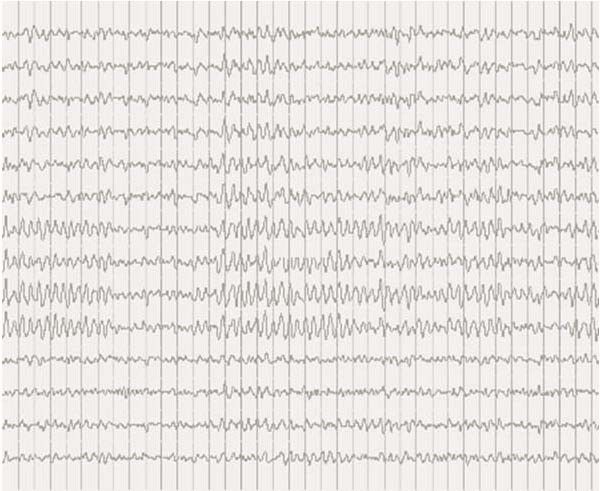
**Ictal recording (during chorea-ballismus episode)**.

Ischaemic and haemorrhagic strokes are the cause of chorea in most elderly patients, and these etiologies were excluded in our patient, based on the neuroimaging studies. Other differential diagnosis considered were: encephalitis, systemic lupus erythematosus, basal ganglia calcifications, Wilson's disease, thyroid disease and tuberous sclerosis. CSF was normal, anti-nuclear antibodies were negative, and serum ceruloplasmin and thyroxine levels were within the normal range. Also, the CT of the head did not reveal any lesion in the basal ganglia, as well as brain MRI exam. EEG was normal, even during the ictal recording, excluding the possibility of epilepsy [Fig. 1, Fig. 2].

Multiple medications in different combinations were tried, including haloperidol, phenobarbital, clonazepam, diazepam, with minimal immediate benefit. Severe pain from the continuous right arm jerking was treated with perfalgan.

Other laboratory tests revealed: haemoglobin of 13 g%, total count of 8,800/mm3, differential count of N 60 E1 L29, and normal liver function tests. Her urea and serum creatinine were 20 mg% and 0.8 mg% respectively. Urine exam: glycosuria, without ketones. Blood glucose concentration was 367 mg% and glycosylated haemoglobin A1C was 13.9%. Estimated blood osmolality was 294 mosm/L and ketones were absent. Serum sodium: 132 mEq/L; potassium: 4.2 mEq/L; serum calcium and magnesium levels were normal. The diagnosis of diabetes was made and the patient was started on rapid analog insulin subcutaneous, until her blood sugar was brought below 250 mg%, following which long-acting insulin was started and continued for the first day. At the end of 24 hours, her blood sugar was 148 mg% and the involuntary movements had completely disappeared. She remained asymptomatic during her hospital stay and was discharged home on a combination of rapid-acting analog, before meals and long-acting analog insulin (subcutaneous) before dinner.

GAD antibodies were positive and C-peptide was low. Based on the presence of glutamic acid decarboxylase antibodies (9,000 U/ml; normal < 1. 4 U/ml), low C-peptide and high insulin requirements, we diagnosed type 1 diabetes mellitus.

## Discussion

The pathogenesis of chorea or ballismus associated with nonketotic hyperglycemia (NKH) is poorly understood. In ketoacidosis, ketones are used as an energy source and GABA can be produced. As a result, HC-HB or partial seizures rarely occur with diabetic ketoacidosis. While in NKH, the brain metabolizes GABA into succinic acid via the succinic acid semialdehyde pathway and thus depletes GABA rapidly. During hyperglycemia, the activity of tricarboxylic acid cycle (Krebs cycle) and glucose utilization are depressed in the brain, so the cerebral metabolism shifts to alternative pathways [3,4]. In nonketotic hyperglycemia, there is a shift to anaerobic metabolism that causes brain to utilise aminobutyric acid which is synthesized from acetoacetate, rapidly depleted, causing cellular dysfunction [5,6]. In addition, striatum may be directly susceptible to alterations in blood glucose levels as shown by paroxysmal choreoathetosis in a patient with hypoglycaemia [7]. Chorea should be considered potentially reversible when associated with nonketotic hyperglycemia, as rapid detection and early correction of hyperglycemia could lead to complete recovery of these involuntary movements in some cases [5,6].

The prognosis of HC-HB as a complication of nonketotic hyperglycemia is excellent. Tight blood glucose control is sometimes sufficient to treat the hemichorea. More often, targeted monotherapy or combination therapy with neuroleptics is also required [6].

Our case illustrates the diagnostic challenges of movement disorders and an association of one with a common medical condition, undiagnosed diabetes, even in the absence of simptomatology secondary to hyperglycemia and glycosuria. Unfamiliarity with these disabling conditions may result in their attribution to psychological or functional disturbances. A high index of suspicion is warranted for neurologic consultation and investigation of patients with abnormal movements.

Epilepsia partialis continua (EPC) is rare, observed in association with cortical lesions of various origins and in some metabolic disorders. EPC as a manifestation of nonketotic hyperglycemia (NKH) was first described in 1965, and cases have been intermittently reported since then [8]. How systemic NKH causes a focal status epilepticus remains unknown. Hypotheses have implicated decreased levels of the inhibitory neurotransmitter gamma aminobutyric acid (owing to inhibition of the Krebs cycle in NKH) or direct effects of hyperglycemia, dehydration or hyperosmolarity on the brain, possibly acting on a previously silent cortical lesion to render it epileptogenic [8-10].

## Conclusion

Severe hyperglycemia without ketosis at the clinical onset of insulin-dependent diabetes mellitus (type 1) has been reported in children and adolescents, but nonketotic hyperglycemia is an unusual cause of chorea-ballismus in children, and chorea-ballismus is also a rare manifestation of primary diabetes mellitus. Since chorea-ballismus can be life-threatening, recognition of this disorder is important because chorea-ballismus caused by hyperglycemia is a treatable disorder with a good prognosis.

## Abbreviations

NKH: nonketotic hyperglycemia; EPC: epilepsia partialis continua; HC-HB: hemichorea-hemiballism; CNS: central nervous system; CSF: cerebrospinal fluid; GAD: glutamic acid decarboxylase (antibodies).

## Consent

Written informed consent was obtained from the patient's mother for the publication of this case report. A copy of the written consent is available for review by the Editor-in-Chief of this journal

## Competing interests

The authors declare that they have no competing interests.

## References

[B1] Zumsteg D, Wennberg RA (2005). A 61-year-old man with continuous clonic jerks of his right leg. CMAJ.

[B2] Oh SH, Lee KY, Im JH (2002). Chorea associated with non-ketotic hyperglycemia and hyperintensity basal ganglia lesion on T1-weighted brain MRI study: a meta-analysis of 53 cases including four present cases. J Neurol Sci.

[B3] Hsu JL, Wang HC, Hsu WC (2004). Hyperglycemia-induced unilateral basal ganglion lesions with and without hemichorea. A PET study. J Neurol.

[B4] Bedwell SF (1960). Some observations on hemiballismus. Neurology.

[B5] Lin JJ, Chang MK (1994). Hemiballism – hemichorea and nonketotic hyperglycemia. J Neurol Neurosurg Psychiatry.

[B6] Rector GW, Herlong HF, Moses H (1982). Nonketotic hyperglycemia appearing as choreoathetosis or ballism. Arch Intern Med.

[B7] Newman RP, Kinkel WR (1984). Paroxysmal choreioathetosis due to hypoglycemia. Arch Neurol.

[B8] Maccario M, Messis CP, Vastola EF (1965). Seizures as manifestations of hyperglycemia without ketoacidosis. Neurology.

[B9] Sanfield JA, Finkel J, Lewis S (1986). Alternating choreoathetosis associated with uncontrolled diabetes mellitus and basal ganglia calcification. Diabetes Care.

[B10] Shan DE, Ho DM, Chang C (1998). Hemichorea – hemiballism; an explanation for MR signal changes. AJNR.

[B11] Lai Ph, Tien Rd, Chang MH (1996). Chorea – ballismus with nonketotic hyperglycemia in primary diabetes mellitus. AJNR.

